# Phyllobilomics: a comprehensive analysis of phyllobilins in apple (*Malus* × *domestica* Borkh.) leaves after fungal infections suggests a general role of the PaO/PB pathway in chlorotic infections

**DOI:** 10.3389/fpls.2026.1876034

**Published:** 2026-08-06

**Authors:** Luca Vestrucci, Lisa Marie Gorfer, Urban Spitaler, Sabine Oettl, Peter Robatscher, Matteo Scampicchio, Michael Oberhuber

**Affiliations:** 1 Laimburg Research Centre, Auer/Ora, Italy; 2 Faculty of Agricultural, Environmental and Food Sciences, Free University of Bozen-Bolzano, Bozen/Bolzano, Italy

**Keywords:** *Alternaria alternata*, *Diplocarpon coronariae*, *Podosphaera leucotricha*, *Venturia inaequalis*, inclusion list, phyllobilins, targeted analysis

## Abstract

**Introduction:**

Chlorophyll (Chl) degradation via the pheophorbide *a* oxygenase/phyllobilin (PaO/PB) pathway produces linear tetrapyrrolic phyllobilins (PBs) during leaf senescence and infection by certain pathogens, such as phytoplasmas. Since leaf yellowing, or chlorosis, is a common symptom of fungal disease in apple (*Malus* × *domestica* Borkh.) and other plants, PB formation was analyzed during four fungal diseases to evaluate the general relevance of the PaO/PB pathway during infection.

**Methods:**

A comprehensive, targeted ultra-high performance liquid chromatography-quadrupole time-of-flight mass spectrometry (UHPLC-QTOF-MS) method was used to profile a total of 79 known and putative PBs in healthy, naturally senescent, and symptomatic leaves infected with *Diplocarpon coronariae, Alternaria alternata, Venturia inaequalis*, or *Podosphaera leucotricha*, using five biological replicates per condition (n=5). Statistical analysis of Chl values was performed using one-way ANOVA followed by Tukey’s post-hoc test.

**Results:**

This method allowed us to detect 36 PBs, including eleven previously unknown PBs, one of which carries an unprecedented methoxy modification. *Diplocarpon coronariae* infection induced extensive chlorosis and a PB profile comparable to senescence, whereas *A. alternata, V. inaequalis*, and *P. leucotricha* did not substantially increase PB diversity or abundance, despite affecting photosynthetic tissues. These results are coherent with the Chl amount in leaves. In *D. coronariae*, an induced extensive chlorosis emerged in infection, with the total Chl reduced from 61.9 ± 5.3 µg cm⁻² in healthy leaves to 12.4 ± 5.3 µg cm⁻² in diseased leaves (p < 0.001, significant reduction), resulting in a PB profile comparable to natural senescence. In contrast, *P. leucotricha* (60.7 vs. 55.5 µg cm⁻²; p = 0.16, no significant difference) and *A. alternata* (41.4 vs. 49.0 µg cm⁻²; p = 0.15, no significant difference), showed no significant reduction in Chl and substantial increase in PB diversity.

**Discussion:**

Our findings indicate that engagement of the PaO/PB pathway in apple leaves is strongly associated with chlorosis, whether induced by natural senescence or specific pathogens, suggesting a general role of this catabolic route in chlorotic infections

## 1 Introduction

**Figure 1:**
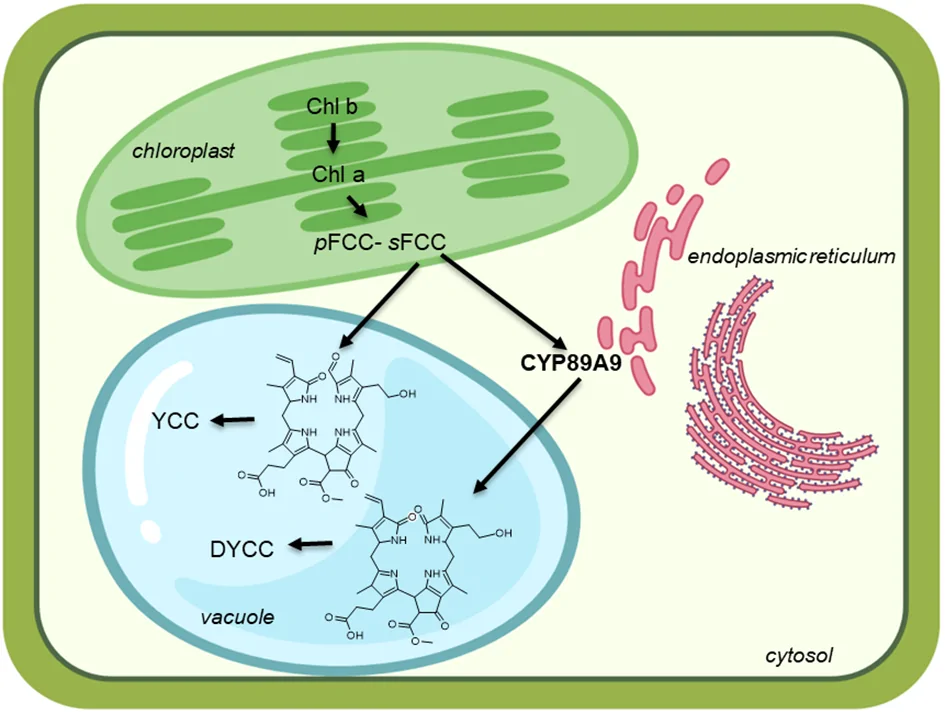
Schematic representation of PB biosynthesis. The process begins in the thylakoids, where Chl *b* is converted to Chl *a*. After the opening of the macrocyclic structure, *p*FCC and *s*FCC are formed. These intermediates are then transported to the cytosol, where both enzymatic and non-enzymatic modifications occur. CYP89A9, located on the membrane of the endoplasmic reticulum, can deformylate FCCs into DFCCs. Subsequently, these compounds accumulate in the vacuole, where acidic conditions isomerize FCCs into NCCs and DFCCs into DNCCs. Further oxidative steps lead to the formation of yellow chlorophyll catabolites (YCCs and DYCCs).

**Figure 2:**
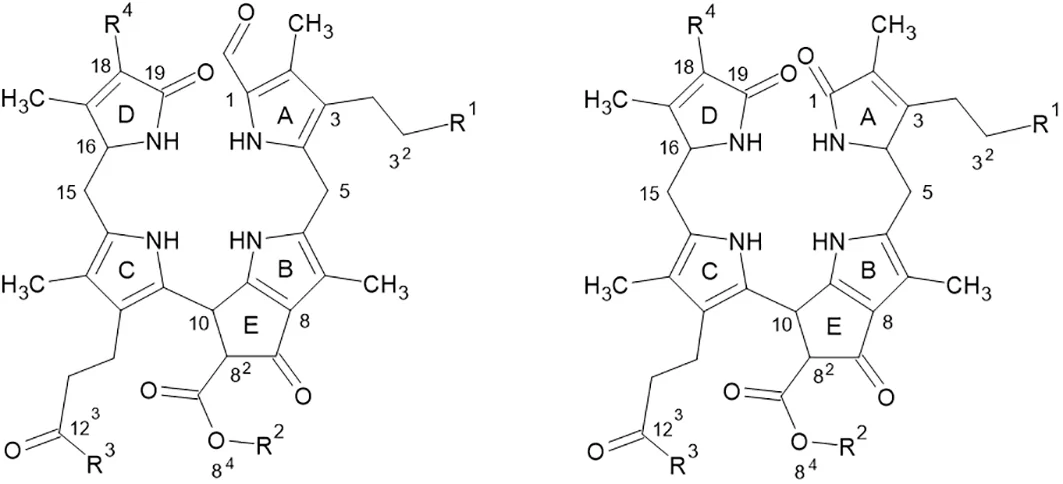
General structure of NCCs (left) and DNCCs (right), showing the position of side chains and atom numbering.

In senescent leaves, chlorophyll (Chl) breakdown proceeds via the pheophorbide *a* oxygenase/phyllobilin (PaO/PB) pathway, producing linear tetrapyrrolic phyllobilins (PBs) that accumulate in vacuoles (Hörtensteiner and Kräutler, 2011; Kräutler, 2014). This metabolic pathway is regulated by phytohormones involved in senescence, including ethylene, jasmonate, and abscisic acid, which enhance the expression of enzymes involved in the PaO/PB pathway through transcription factors (Hörtensteiner et al., 2019). Moreover, these phytohormones play a role in regulating defense responses in plants (Kachroo and Kachroo, 2007). PaO opens the cyclic structure of Chl to yield red chlorophyll catabolite (RCC), the first linear tetrapyrrolic product (Hörtensteiner et al., 2019). Subsequently, RCC is reduced by RCC reductase, which forms fluorescent chlorophyll catabolite (FCC) (Hörtensteiner et al., 2019). FCC and its corresponding dioxobilin-type FCC (DFCC), obtained by a deformylation process catalyzed by CYP89A9 in cytosol, isomerize in the acidic milieu of the vacuole, forming diverse chemical species (Kuai et al., 2018), including non-fluorescent chlorophyll catabolites (NCCs), dioxobilin-type catabolites (DNCCs), and their oxidized derivatives (**Figures 1**, **2**). The comprehensive, targeted investigation of PBs, termed phyllobilomics, applies advanced metabolomics principles to comprehensively profile Chl catabolites. To date, at least 73 structurally characterized phyllobilins have been isolated across more than 30 plant species. They differ primarily in (i) the oxidation state along the tetrapyrrolic backbone, (ii) the configuration of stereocenters, and (iii) the functionalization of side chains extending from the pyrrole rings (Karg et al., 2023). As constituents of plant foods, PBs have documented physiological activities *in vitro* and in cells, including potent antioxidant and free-radical–scavenging capacity and protection against oxidative stress, with activities comparable to those of bilirubin (Müller et al., 2007; Ulrich et al., 2011; Karg et al., 2019). While this pathway is well characterized during natural senescence, its role during biotic stress, particularly fungal infection, is less understood. In some diseases, chlorosis suggests an extensive engagement of the PaO pathway, whereas in others, necrotic lesions or subtle discoloration may indicate the onset of Chl breakdown or alternative stress responses (Müller et al., 2007; Ulrich et al., 2011; Karg et al., 2019, 2023). In plants, the physiological roles of PBs remain less well established beyond their involvement in Chl detoxification and contributions to visible pigmentation. Previous research detected PBs in phytoplasma-infected apple and apricot leaves, implicating activation of the PaO/PB pathway during disease development (Mittelberger et al., 2017). Until recently, a key bottleneck was the lack of a single liquid chromatography-mass spectrometry (LC-MS) method able to broadly screen for all known PBs; this has been addressed by ultra-high performance liquid chromatography-quadrupole time-of-flight mass spectrometry (UHPLC-QTOF-MS) workflows with MS^2^-based identification and an inclusion list of 79 PB candidates. This list includes 73 previously detected PBs and six potential candidates based on structural analogies and biosynthetic relationships within the PB pathway.

Apple (*Malus × domestica* Borkh.) is one of the most widely cultivated fruit crops worldwide, with an annual production of more than 87 million metric tons in 2019 (Duan et al., 2021). Its economic importance is threatened by several fungal diseases that cause substantial yield losses and quality reductions, including apple scab (*Venturia inaequalis* (Cooke) G. Winter), powdery mildew ((*Podosphaera leucotricha* (Ellis & Everh.)), apple blotch Jørst., syn. *Marssonina coronaria* (Ellis & J.J. Davis) Jørst., and Alternaria leaf blotch (*Alternaria alternata* (Fr.) Keissl).

Apple blotch, caused by *D. coronariae*, primarily affects mature leaves, often close to harvest (Cheng et al., 2021). Another disease that causes visible modifications in leaves is leaf blotch, caused by *A. alternata* (Gur et al., 2017; Tian et al., 2024). Disease symptoms on leaves are characterized by brown spots, which occur mainly between spring and early summer (Li et al., 2013). The causal agent responsible for apple scab is *V. inaequalis*. Symptoms include scattered, olivaceous green, chlorotic, sporulating lesions on the infected leaf surface (Xu et al., 2008; Bowen et al., 2011). Infections not only affect the appearance of the leaves but also the photosynthetic apparatus (Bowen et al., 2011). Symptoms of powdery mildew, caused by *P. leucotricha*, typically appear on the underside of leaves, where infected leaves curl longitudinally, displaying mycelium and spores that form characteristic white patches (Strickland et al., 2021). The fungus causes significant damage to trees by reducing photosynthetic activity (Ellis, 1981) and inducing minor chlorotic areas on the leaf surface (Strickland et al., 2020).

To investigate the regulation of the PaO/PB pathway in an agronomically major fruit crop, we applied our analytical approach to apple as a model system, categorized by their symptom phenotype: (i) those associated with distinct chlorotic lesions (e.g., *Diplocarpon coronariae* (Ellis & J.J. Davis)) and (ii) those eliciting predominantly necrotic or subtle symptom patterns (e.g., *Alternaria alternata* (Fr.) Keissl, *Venturia inaequalis* (Cooke) G. Winter, and *Podosphaera leucotricha* (Ellis & Everh.) E.S. Salmon). The results were used to clarify the relationship between visible chlorosis, Chl degradation, and PB diversity, and to evaluate PBs as potential biomarkers of biotic stress in apple leaves.

## 2 Material and methods

### 2.1 Sample collection

Leaf samples from the Golden Delicious, Gala, and Red Delicious cultivars (*Malus* × *domestica* Borkh.) were collected from apple orchards cultivated according to Italian integrated pest management guidelines (**Table 1**). Sampling started in July 2022 and continued until December 2022, as shown in **Table 1**. Climatic conditions during the 2022 sampling in South Tyrol, Italy, varied significantly. The July pathogen collection was characterized by warm, dry weather (mean: 27 °C–28 °C; maximum: 35 °C–39 °C), whereas the late November senescence and early December collection periods were marked by a sharp cooling trend (mean: 2 °C–5 °C; minimum: -3 °C; relative humidity: >80%). Healthy and diseased samples were collected on the same day, whereas senescent apple leaves were collected in autumn and early winter, when signs of senescence were visible. For each of the three conditions (healthy, diseased, and senescent), five individual trees were selected, and 10 leaves were collected from each tree. Samples were collected from the outer, well-exposed middle section of the canopy (approximately 1.5 m above the ground) to ensure physiological uniformity. Five leaves from each of the five individual trees (25 leaves per condition, representing five independent biological replicates, n = 5) were selected for Chl quantification and PB extraction. Leaf samples infected with *V. inaequalis* (cv. Golden Delicious), *P. leucotricha* (cv. Gala), and *A. alternata* (cv. Golden Delicious) were collected from orchards at the Laimburg Research Center at the following coordinates: (46.383909, 11.291117), (46.3844079, 11.2913194), and (46.3869614, 11.2896538), respectively. All diseased leaves were symptomatic, with characteristic chlorotic spots on the leaf surface. Due to the sampling approach, a visual disease severity scale was not recorded; instead, Chl (*a*, *b*, and total) content was used as an objective and quantitative physiological indicator of infection intensity. Samples for *D. coronariae* (cv. Red Delicious) were collected from an orchard located in Kuens/Caines (Italy), at the following coordinates: (46.693382, 11.174718). Infected leaves were in a premature senescence stage caused by the disease. Natural leaf infections were achieved by omitting the use of fungicides effective against the respective pathogens. Leaves were stored at –80 °C prior to analysis. Samples were labeled with a code indicating the plant matrix (*Md*, *Malus* × *domestica*), a progressive number, and the condition (H = healthy, D = diseased, and S = senescent), as shown in **Table 1**. For PCR analysis, 10 leaves per disease were collected separately.

**Table 1:** Plant diseases and sample set.

Pathogen	Disease	Disease symptoms	Apple cultivar	Location	Altitude (a.s.l.)	Sampling date (2022)	Samples
*Diplocarpon coronariae*	Marssonina apple blotch	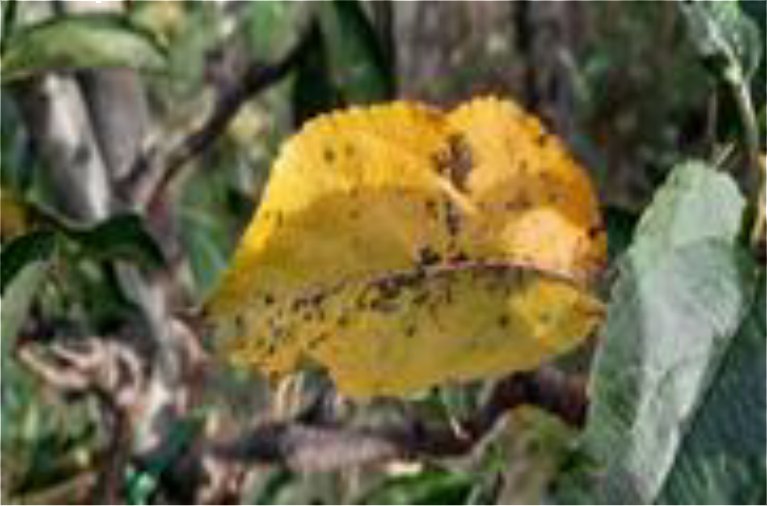	Red Delicious	Kuens/Caines	455 m	September 27 (H+D) December 7 (S)	*Md*_16-20_H *Md*_16-20_D *Md*_16-20_S
*Alternaria alternata*	Alternaria leaf blotch	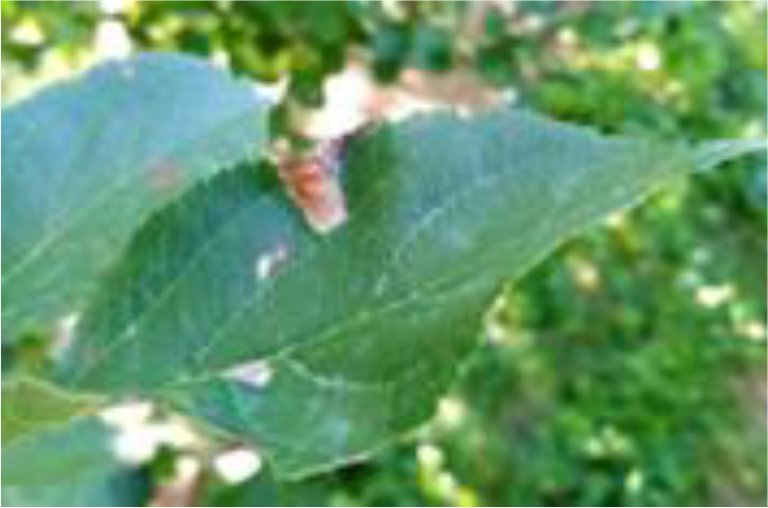	Golden Delicious	Laimburg	224 m	July 29 (H+D) November 25 (S)	*Md*_11-15_H *Md*_11-15_D *Md*_11-15_S
*Venturia inaequalis*	Apple scab	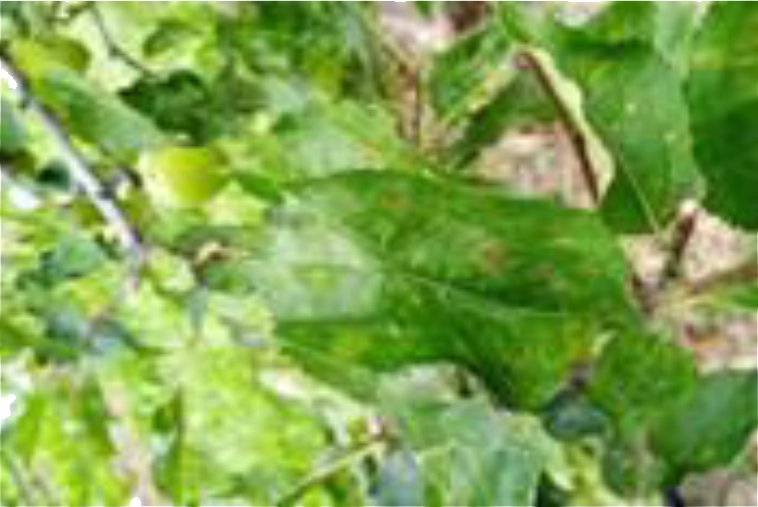	Golden Delicious	Laimburg	224 m	July 14 (H+D) November 25 (S)	*Md*_1-5_H *Md*_1-5_D *Md*_1-5_S
*Podosphaera leucotricha*	Powdery mildew	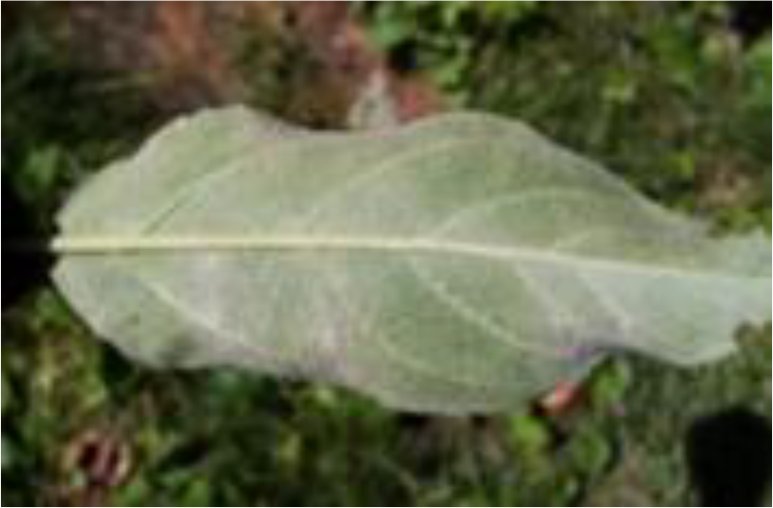	Gala	Laimburg	224 m	July 29 (H+D) November 25 (S)	*Md*_6-10_H *Md*_6-10_D *Md*_6-10_S

Apple cultivar, sampling location, and sample information are provided (*Md*, *Malus × domestica*; H, healthy; D, diseased; S, senescent).

### 2.2 Identification of pathogens

Infected leaves were accurately identified based on disease symptoms and confirmed through molecular analysis. DNA was extracted from symptomatic apple leaves using the Quick-DNA™ Tissue/Insect Miniprep Kit (Zymo Research, CA, USA). PCR was performed in a 35 μL reaction volume using a thermal cycler (Veriti™ 96-Well Fast Thermal Cycler, Thermo Fisher Scientific Inc., MA, USA). The obtained PCR products were purified with the QIAquick PCR Purification Kit (Qiagen, Hilden, Germany) and sequenced by LGC Genomics GmbH (Berlin, Germany). Quality control of sequenced data was performed with Geneious 11.1.5 software. Sequence identity was confirmed using the BLASTN algorithm at NCBI GenBank (https://www.ncbi.nlm.nih.gov/genbank/). For *P. leucotricha*, molecular identification involved amplification of the mitochondrial cytochrome *b* gene using the primer pair PM-COB-for (5′-ACAGCTTCAGCTTTCTTCTTTTTAG-3′) and PM-COB-rev (5′-ATTCATGGTATTGCACTCATAAGGT-3′) (Lesemann et al., 2006). For *V. inaequalis*, the ITS region was amplified using the primer pair ITS4 (5′-TCCTCCGCTTATTGATATGC-3′) and Vina (5′-GTCTGAGAACAAGTTAAATAA-3′) (White et al., 1990; Stehmann et al., 2001). For both *D. coronariae* and *A. alternata*, molecular identification and amplification of the ITS region was performed using the primer pair ITS4 (5′-TCCTCCGCTTATTGATATGC-3′) and ITS6 (5′-GAAGGTGAAGTCGTAACAAGG-3′) (White et al., 1990; Cooke et al., 2000).

### 2.3 Chlorophyll extraction

Five leaves per sample were individually extracted and analyzed (**Supplementary Figure 1**). For each leaf, two leaf disks (diameter, 1 cm) were obtained from each side of the midrib and placed into two 2.5 mL Eppendorf^®^ tubes for extraction. A spatula tip of CaCO_3_ (puriss., Sigma-Aldrich, Milan, Italy) was added (approximately 2.0 ± 0.1 mg), and leaf disks were frozen in liquid nitrogen. Samples were ground using a MM400 mixer mill (Retsch, Haan, Germany) for 30 s at 30 Hz. Extraction was performed using LC-MS grade methanol (Honeywell Riedel-de Haen, Charlotte, NC). First, 1 mL of methanol was added, followed by vortexing and shaking in an Eppendorf^®^ thermomixer (10 min at 4 °C and 14,000 rpm). The sample was then centrifuged (10 min at 4 °C and 14,000 rpm). This process was repeated twice, with 0.5 mL of methanol added during the final step. Methanolic extracts from both tubes were then combined and transferred into a 15 mL Falcon^®^ tube. The solution was centrifuged again (10 min at 4 °C and 14,000 rpm). The volume was adjusted to 5 mL with methanol, and an aliquot of the supernatant was collected to determine Chl content by UV–VIS measurements using a Cary 60 spectrophotometer (Agilent Technologies, Santa Clara, CA). The remaining extract was stored at –80 °C for PB analysis.

### 2.4 Chlorophyll amount

An aliquot of 0.2 mL of the supernatant was used for healthy and diseased leaves. For leaves infected with *D. coronariae*, which exhibited extensive chlorosis, and for all senescent leaves, 0.5 mL of supernatant was used. Methanol was added to obtain a final volume of 1 mL in the cuvettes, and Chl content was measured by UV–Vis spectrometry, according to Porra et al. (1989). The contents of Chl *a*, Chl *b*, and total Chl (Chl *a* + Chl *b*) were calculated by applying the Lambert−Beer law at three wavelengths (665.2, 650.0, and 750.0 nm). For each sample, five leaves were analyzed.

### 2.5 Qualitative PB analysis

A quadrupole-time-of-flight mass spectrometer (UHPLC–HRMS-Q–TOF) IMPACT HD (Bruker, Bremen, Germany) was used to identify PBs. The instrument was coupled with an UltiMate 3000 RSLC pump, an online degasser, a binary pump, a column oven, and a 3000RS DAD detector, all from Thermo Fisher Scientific (Waltham, MA). The appropriate precolumn was an Acquity UPLC BEH C18 VanGuard: 130 Å, 1.7 µm, 2.1 mm X 5 mm, and 3/pk (Waters, Milford, MA). The column was an Acquity UPLC BEH C18: 130Å, 1.7 µm, 2.1 mm X 150 mm, and 1/pk (Waters). The column oven temperature was set to 25 °C. A pure NCC-644 standard, isolated from *Cercidiphyllum japonicum* leaves, was provided by the University of Innsbruck (Austria). A quantity of 2.5 mL of the Chl extract was transferred to a 15 mL Falcon^®^ tube, centrifuged (4,000 rpm, 4 °C, 10 min) and dried using Concentrator plus/Vacufuge^®^ plus (Eppendorf, Hamburg, Germany) in V-AL mode at room temperature (RT) for 1 h. If the solvent was not completely evaporated, the extracts were stored at –80 °C before repeating this procedure. To the Falcon^®^ tube, 0.5 mL of MeOH:buffer (1:1 mixture of LC-MS grade methanol and 4 mM NH_4_OAc, purchased from Honeywell Riedel-de Haen, Charlotte, NC) was added, followed by vortexing and centrifugation (10 min at 4 °C and 14,000 rpm). The solution was then transferred to a 2-mL microcentrifuge tube. This step was repeated once more, and the extracts were combined. Afterwards, the centrifuged solution was passed through a PTFE 0.2 μm filter and stored at –80 °C until analysis.

Analysis of PBs was conducted using UHPLC–HRMS-Q–TOF under the following conditions: an injection volume of 20 μL and a flow rate of 0.3 mL min^-1^. The mobile phases consisted of solvent A, 4 mM NH_4_OAc at pH 7, and solvent B, acetonitrile (ACN, LC-MS grade, purchased from Honeywell Riedel-de Haen, Charlotte, NC). The gradient was set as follows: 0 min, 10% B; 0–6 min, 10–30% B; 6–7.65 min, 30–70% B; 7.65–8.3 min, 70–95% B; 16 min, 95% B; 16–19 min, 95%–10% B; 21 min, 10% B. All parameters were set according to Gorfer et al. (2022a). The ESI source was operated in positive mode with an end plate offset of 500 V, a capillary voltage of 4,500 V, a nebulizer pressure of 3.0 bar, a dry gas flow rate of 12.0 L min^-1^, and a dry gas temperature of 230 °C. A sodium formate solution (10 mM) was used as an internal standard. The acquisition ranged from 50 *m/z* to 1,500 *m/z* at a spectral rate of 0.5 Hz, using both full-scan and MS^2^ modes (Gorfer et al., 2022a). An inclusion list of all known and hypothesized pseudo-molecular ions of PBs (79 in total) was used in the MS^2^ method, with a mass width tolerance set to 3.0 *m*/*z*. The number of precursors was set to three, with a cycle time of 3.0 s and a minimum intensity threshold of 4,285 counts. Dynamic exclusion was configured to exclude a precursor after two spectra and release it after 0.20 min. Collision energy was set to 30, 35, 40, and 45 eV. PB analysis was performed by randomly injecting leaf sample extracts, blanks (MeOH), and a PB standard (NCC-644). All data were analyzed using the Bruker Compass DataAnalysis 4.2 software. PBs were confirmed based on retention time, molecular ions, and mass fragments by analyzing extracted ion chromatograms (EICs) at their respective *m/z* values (± 0.01). PBs were identified according to a three-tier confidence scheme (Reisdorph et al., 2019). While NCC-644 was confirmed using an authentic external standard (Tier 1), all other endogenous compounds were detected at Tier 2 and Tier 3 confidence levels. Tier 2 comprised putatively annotated compounds based on accurate mass and a complete set of literature MS^2^ diagnostic fragment ions. Tier 3 comprised tentatively characterized compounds, including newly described PBs lacking previous literature reference data and/or signals with partial MS^2^ (e.g., NCC-674) evidence due to very low intensity, where structural alternatives could not be excluded.

### 2.6 Statistical analysis

Statistical analysis of total Chl content in leaf samples under each condition (healthy, diseased, and senescent) for the four diseases investigated was performed using one-way ANOVA followed by Tukey’s *post-hoc* test using R (R Core Team, Vienna, Austria), with a significance level of p ≤ 0.05. ANOVAs were performed separately for each disease. **Figure 1** was created using BioRender.com.

To evaluate the qualitative similarity of the PB profiles between diseased leaves and the senescent reference group, Jaccard’s similarity index was applied to the presence/absence data, where a value of 100% indicates complete overlap and 0% indicates no shared compounds.

## 3 Results and discussion

### 3.1 Molecular identification of fungal pathogens

Pathogens were unambiguously identified using DNA sequencing. For *P. leucotricha*, the obtained sequence showed 98.4% identity to NCBI GenBank accession number DQ534067 and was deposited under accession number PQ557569. For *V. inaequalis*, the sequence showed 100% identity to EU282477 (Sánchez-Torres et al., 2009) and was deposited under accession number PQ526747. The obtained *D. coronariae* sequence showed 99.6% identity to OR241491 and was deposited under accession number PQ526748. The obtained *A. alternata* sequence showed 100% identity to KY996469 and was deposited under accession number PQ526749.

### 3.2 Chlorophyll concentration in healthy, diseased, and senescent apple leaves

**Figure 3:**
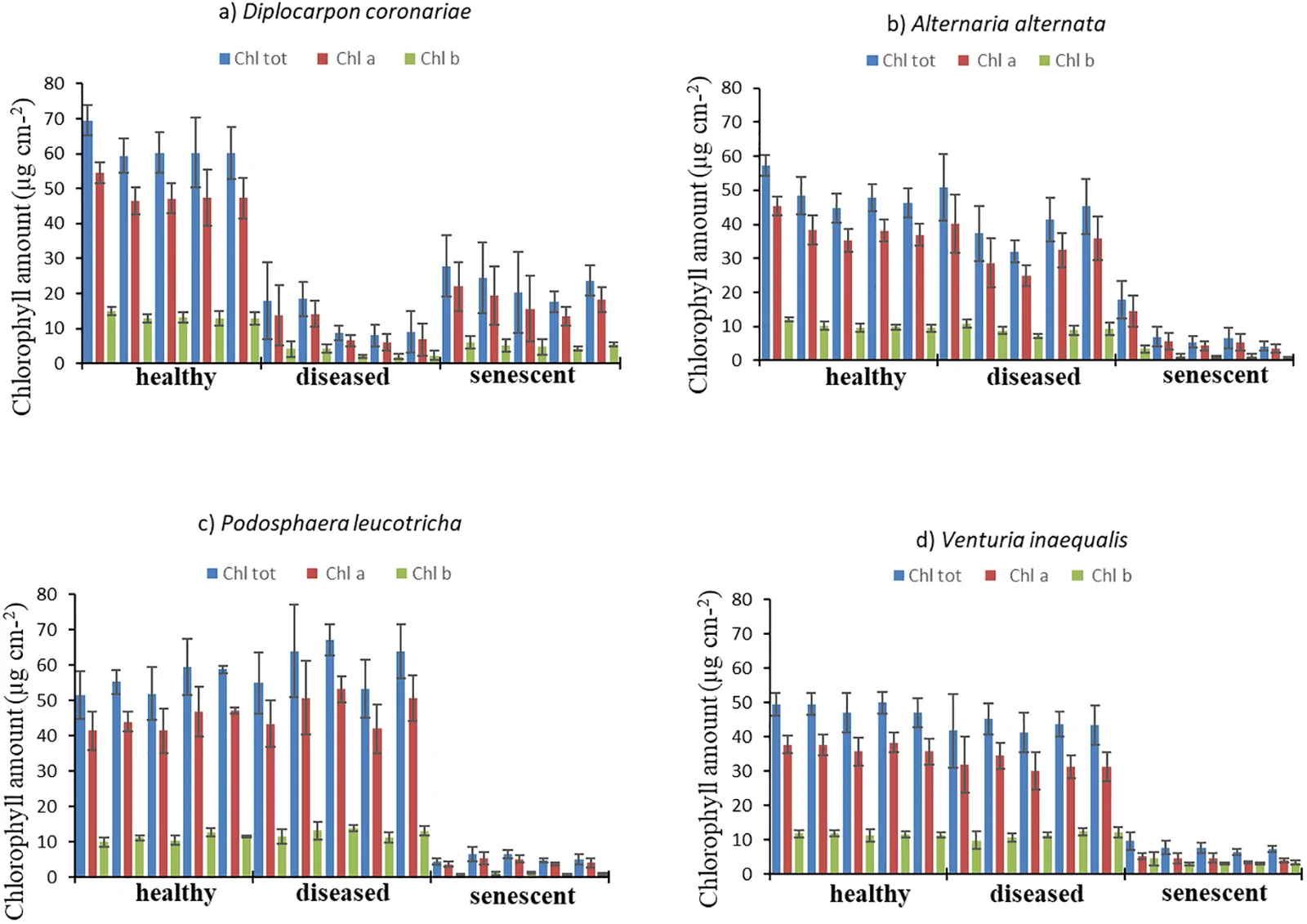
Clustered column charts showing chlorophyll levels in healthy, diseased, and senescent leaf samples across different pathogens: **(a)** *Diplocarpon coronariae*, **(b)** *Alternaria alternata*, **(c)** *Podosphaera leucotricha*, and **(d)** *Venturia inaequalis*. For each sample, total chlorophyll (Chl tot), Chl a, and Chl b, together with their respective standard deviations (n = 5) are reported.

Five leaves from each replicate were used for the determination of Chl concentration. Results for all samples, including total Chl, Chl *a*, and Chl *b*, are shown in **Figure 3** and listed in **Supplementary Table 1**. An ANOVA was used to compare sample groups for all four diseases. As expected, total Chl concentrations in healthy leaves were comparable among all samples, ranging between 50 and 70 µg cm^-^². Uninfected senescent leaves showed total Chl values of approximately 10 µg cm^-^² after Chl degradation, consistent with values reported in the literature (Mattila et al., 2021).

One notable exception was the senescent leaves of cv. Red Delicious, used for the *D. coronariae* infection, which exhibited total Chl concentrations of 18–28 µg cm^-^² (**Figure 3**). This finding might be related to incomplete Chl degradation, even though the senescent leaves were collected on 7 December 2022. To validate their physiological status, we analyzed the Chl *a*/Chl *b* ratio. During a canonical senescence pathway, Chl *a* is preferentially degraded, causing a marked decline in the ratio. In the senescent cv. Red Delicious leaves collected in December; however, the Chl *a*/Chl *b* ratio remained anomalously high, averaging 3.50, and thus confirmed an incomplete or cold-arrested senescence process. Infection of green leaves of this cultivar by *D. coronariae* resulted in extensive chlorosis (**Table 1**), indicative of Chl degradation; correspondingly, Chl content was reduced to 7.95–18.44 µg cm^-^² (mean = 12.37 µg cm^-^², SD = ± 5.31 µg cm^-^², **Figure 3**). Although leaves were selected to be visually homogeneous in terms of extensive chlorotic symptoms, this variance highlights the independent and dynamic progression of the fungal infection within individual biological replicates. Since a standardized visual disease severity scale was not recorded during sampling, Chl content served as a parameter to indicate disease progression and the trend of PB formation. Infected leaves exhibited small black-purple necrotic lesions on their upper surface, accompanied by irregular patterns and circular spots, while the entire leaf showed a yellowish discoloration. This pathogen disrupts the photosynthetic apparatus, leading to visibly yellow leaves on affected trees during summer, suggesting Chl breakdown due to infection.

Similarly, *V. inaequalis*-infected leaves (cv. Golden Delicious) contained significantly less Chl than healthy leaves, even though they showed only faint symptoms of chlorosis. For this group, senescent leaves reached very low Chl concentrations (F_2_,_12_ = 1280.99, p < 0.001). Likewise, for *D. coronariae* (cv. Red Delicious), which induced the most dramatic chlorophyll reduction among the pathogen groups, total Chl contents were significantly lower than in healthy controls (F_2_,_12_ = 165.03, p < 0.001; Tukey *post-hoc* diseased vs. healthy, p_adj_ < 0.001). In the case of *P. leucotricha* (cv. Gala) and *A. alternata* (cv. Golden Delicious) infections, symptomatic leaves and healthy leaves could not be distinguished significantly by their total Chl content, even though there was considerable variation. Again, senescent leaves showed low total Chl levels (**Figure 3**, F_2_,_12_ = 266.7, p < 0.001). Overall, the Chl contents in healthy and senescent leaves were consistent with literature values for other plant species. In addition, they were in line with visual signs of chlorosis (Li et al., 2018; Mattila et al., 2021).

### 3.3 Identification of PBs in healthy, diseased and senescent apple leaves

The distribution of PBs across the individual leaf extracts was evaluated by UHPLC–HRMS-Q–TOF screening using an inclusion list of 79 candidates. Compounds were identified based on their retention times and MS matches with high mass accuracy (5 ppm). PBs were named according to their chemical class and neutral mass (**Table 2**).

**Table 2:** List of PBs detected in this study in full-scan and MS^2^ modes.

Entry number	PB reported as	Denomination	Formula	Theoretical *m/z* (M+H)^+^	References	Tier	Side chains (see Figure 2)			
							R1 (C3^2^)	R2 (O8^4^)	R3 (C12^3^)	R4 (C18)
1	Pheo *a*	Pheo *a*	C_35_H_36_N_4_O_5_	593.2758	(Viera et al., 2018)	2	H	CH_3_	OH	CHCH_2_
2	YCC-612	YCC-612	C_34_H_36_N_4_O_7_	613.2656		3	H	H	OH	CHCH_2_
3	*At*-mes16-DNCC-47	DNCC-616	C_34_H_40_N_4_O_7_	617.2970	(Süssenbacher et al., 2014)	2	H	CH_3_	OH	CHCH_2_
4	*Cj*-YCC-2	YCC-626	C_35_H_38_N_4_O_7_	627.2813	(Moser et al., 2008)	2	H	CH_3_	OH	CHCH_2_
5	YCC-628	YCC-628	C_34_H_36_N_4_O_8_	629.2605		3	OH	H	OH	CHCH_2_
6	*Cj*-NCC-2/*So*-NCC-5/*Md*-NCC-58/*Oe*-NCC-3/*Pa*-NCC-58/*Pd*-NCC-71	NCC-628	C_35_H_40_N_4_O_7_	629.2970	(Mittelberger et al., 2017; Erhart et al., 2016)	2	H	CH_3_	OH	CHCH_2_
7	*At-*NCC-3	NCC-630	C_34_H_38_N_4_O_8_	631.2762	(Pružinská et al., 2005)	2	OH	H	OH	CHCH_2_
8	*Pp*-DNCC/*Hvir*-DNCC/*Ej*-DNCC-1/*At*-mes16-DNCC-38/*Vv*-DNCC/*Md*-DNCC-632	DNCC-632	C_34_H_40_N_4_O_8_	633.2919	(Gorfer et al., 2022a; Ríos et al., 2014b; Curty and Engel, 1996; Ríos et al., 2015; Moser et al., 2012; Das et al., 2018)	2	OH	CH_3_	OH	CHCH_2_
9	DNCC-636	DNCC-636	C_33_H_40_N_4_O_9_	637.2868		3	H	H	OH	CH(OH)-CH_2_OH
10	PiCC-640	PiCC-640	C_35_H_36_N_4_O_8_	641.2606	(Mittelberger et al., 2017)	2	OH	CH_3_	OH	CHCH_2_
11	*Cj*-YCC-1/*Md*-YCC-54/*Pa*-YCC-54/*Po*-YCC/*Ep*-YCC-6/*Ps*-YCC-1	YCC-642	C_35_H_38_N_4_O_8_	643.2762	(Mittelberger et al., 2017; Gorfer et al., 2022a; Erhart et al., 2016; Roca et al., 2017)	2	OH	CH_3_	OH	CHCH_2_
12	*Cj*-NCC-1/*Lo*-NCC-1/*Ls*-NCC-1/*Ms*-NCC-2/*Pc*-NCC-2/*So*-NCC-4/*Sw*-NCC-58/*Md*-NCC-2/*Mc*-NCC-61/*Ej*-NCC-4/*Pd*-NCC-60/*Ps*-NCC-3/*Md*-NCC-50/*Oe*-NCC-2	NCC-644	C_35_H_40_N_4_O_8_	645.2919	(Mittelberger et al., 2017; Müller et al., 2007; Gorfer et al., 2022a; Curty and Engel, 1996; Roca et al., 2017)	1	OH	CH_3_	OH	CHCH_2_
13	YCC-646	YCC-646	C_34_H_38_N_4_O_9_	647.2717		3	H	H	OH	CH(OH)-CH_2_OH
14	*Cl*-NCC-3	NCC-648	C_34_H_40_N_4_O_9_	649.2868	(Ríos et al., 2015)	3	H	H	OH	CH(OH)-CH_2_OH
15	*Mc*-*hm*FCC-71	hmFCC-658	C_36_H_42_N_4_O_8_	659.3075	(Moser et al., 2012)	3	OH	CH_3_	OCH3	CHCH_2_
16	YCC-660	YCC-660	C_35_H_40_N_4_O_9_	661.2868		3	H	CH_3_	OH	CH(OH)-CH_2_OH
17	*Ta*-NCC-662	NCC-662	C_35_H_42_N_4_O_9_	663.3025	(Das et al., 2018)	3	H	CH_3_	OH	CH(OH)-CH_2_OH
18	*So*-NCC-1	NCC-664	C_34_H_40_N_4_O_10_	665.2817	(Berghold et al., 2002)	3	OH	H	OH	CH(OH)-CH_2_OH
19	*Co*-DNCC-2/*Ap*-DNCC/*Hv*-DNCC/*Md*-DNCC-666	DNCC-666	C_34_H_42_N_4_O_10_	667.2974	(Gorfer et al., 2022a; Ríos et al., 2014a; Müller et al., 2011)	2	OH	CH_3_	OH	CH(OH)-CH_2_OH
20	YCC-672	YCC-672	C_36_H_40_N_4_O_9_	673.2868		3	OH	CH_3_	OH	CHCH_2_
21	OMe-*Cj*-NCC-1	NCC-674	C_36_H_42_N_4_O_9_	675.3025		3	OH	CH_3_	OH	CHCH_2_
22	*Ep*-YCC-3	YCC-676	C_35_H_40_N_4_O_10_	677.2817	(Karg et al., 2019b)	2	OH	CH_3_	OH	CH(OH)-CH_2_OH
23	*So*-NCC-2/*Mc*-NCC-42/*Ej*-NCC-1/*Pd*-NCC-40/*Md*-NCC-35/*Hv*-NCC-1/*Pa*-NCC-35	NCC-678	C_35_H_42_N_4_O_10_	679.2973	(Mittelberger et al., 2017; Gorfer et al., 2022a; Ríos et al., 2014b; Erhart et al., 2016; Moser et al., 2012; Berghold et al., 2002)	2	OH	CH_3_	OH	CH(OH)-CH_2_OH
24	NCC-692	NCC-692	C_36_H_44_N_4_O_10_	693.3130		3	OH	CH_3_	OCH_3_	CH(OH)-CH_2_OH
25	YCC-728	YCC-728	C_38_H_40_N_4_O_11_	729.2766		3	O-Mal	CH_3_	OH	CHCH_2_
26	YCC-790	YCC-790	C_40_H_46_N_4_O_13_	791.3134		3	O-Glc	H	OH	CHCH_2_
27	*Bn*-NCC-2/*At*-NCC-1/*Bo*-NCC-1	NCC-792	C_40_H_48_N_4_O_13_	793.3291	(Roiser et al., 2015; Pružinská et al., 2005; Mühlecker and Kräutler, 1996)	3	O-Glc	H	OH	CHCH_2_
28	*Ep*-YCC-5/*Ps*-YCC-2	YCC-804	C_41_H_48_N_4_O_13_	805.3290	(Gorfer et al., 2023a; Roca et al., 2017)	2	O-Glc	CH_3_	OH	CHCH_2_
29	*Nr*-NCC-2/*Zm*-NCC-2/*Pc*-NCC-1/*Ms*-NCC-1/*Tc*-NCC-2/*Mc*-NCC-59/*Pd*-NCC-56/*Ug*-NCC-43/*Ps*-NCC-2/*Md*-NCC-47/*At*-NCC-4/*Md*-NCC-1/*Pa*-NCC-47	NCC-806	C_41_H_50_N_4_O_13_	807.3447	(Müller et al., 2007; Mittelberger et al., 2017; Gorfer et al., 2022a; Berghold et al., 2004; Scherl et al., 2012; Moser et al., 2012; Scherl et al., 2016; Roca et al., 2017)	2	O-Glc	CH_3_	OH	CHCH_2_
30	*Co*-NCC-826	NCC-826	C_40_H_50_N_4_O_15_	827.3345	(Gorfer et al., 2023a; Ríos et al., 2014a)	3	O-Glc	H	OH	CH(OH)-CH_2_OH
31	*Co*-DNCC-1/*Md*-DNCC-828	DNCC-828	C_40_H_52_N_4_O_15_	829.3502	(Gorfer et al., 2022a; Erhart et al., 2016)	3	O-Glc	CH_3_	OH	CH(OH)-CH_2_OH
32	*Ep*-YCC-2	YCC-838	C_41_H_50_N_4_O_15_	839.3345	(Karg et al., 2019b)	3	O-Glc	CH_3_	OH	CH(OH)-CH_2_OH
33	*Zm*-NCC-1/*Tc*-NCC-1/*Co*-NCC-1/*Pd*-NCC-35/*Md*-NCC-31/*Pa*-NCC-31/*Pa*-NCC-33/*Ug*-NCC-27	NCC-840	C_41_H_52_N_4_O_15_	841.3502	(Mittelberger et al., 2017; Gorfer et al., 2022a; Erhart et al., 2016; Ríos et al., 2014a; Scherl et al., 2012; Berghold et al., 2006)	2	O-Glc	CH3	OH	CH(OH)-CH2OH
34	*Md*-DNCC-990	DNCC-990	C_46_H_62_N_4_O_20_	991.4030	(Gorfer et al., 2022a)	3	O-Glc	CH_3_	OH	CH(OH)-CH_2_O-Glc
35	YCC-1000	YCC-1000	C_47_H_60_N_4_O_20_	1001.3873		3	O-Glc	CH_3_	OH	CH(OH)-CH_2_O-Glc
36	*Pd-*NCC-32/*Md*-NCC-29/*Pa*-NCC-29	NCC-1002	C_47_H_62_N_4_O_20_	1003.4030	(Mittelberger et al., 2017; Gorfer et al., 2022a; Erhart et al., 2016)	2	O-Glc	CH_3_	OH	CH(OH)-CH_2_O-Glc

The table reports molecular formulae, theoretical masses, confidence levels of identification (Tiers 1–3), and peripheral substituents. Previously reported PBs are annotated with their corresponding literature references.

Notably, the precursor of PBs, Pheo *a* (C_3_H_36_N_4_O_5_), an apolar Chl breakdown intermediate with an intact chlorin macrocycle (Hörtensteiner et al., 2019), was identified in all samples. Typical fragments, as illustrated by Viera et al. (2018), allowed the identification of this molecule. Following the pathway architecture described by Hörtensteiner et al. (2019), the primary metabolic divergent point resides at or after the PaO enzymatic step. However, it is worth noting that while Pheo a levels reflect this metabolic baseline, a minor fraction of the detected Chl in green tissues might also derive from unavoidable, minor *ex vivo* pheophytinization of chlorophyll during the methanol-based extraction process. Indeed, even with an extraction efficiency exceeding 90% in methanol, a small portion (up to 10%) of the total Chl can undergo transformation or degradation into derivative forms during solvent isolation (Sitarek-Andrzejczyk et al., 2026). A total of 36 PBs were detected in this study across all samples, spanning healthy, senescent, and pathogen-infected leaves. While these catabolites were mostly concentrated in senescent tissues, our findings provide the most comprehensive description of PBs in apple leaves under both physiological and pathological conditions, achieved through a recently published high-resolution LC–MS method (Gorfer et al., 2022a). Using this workflow, a total of 13 PBs already known to occur in apple were identified, 12 PBs known from other plant species were identified in apple for the first time, and 11 PBs were tentatively identified for the first time, including one compound with an unprecedented addition of a methoxy moiety (see below). The identification of PBs and the characterization of their side-chain substituents, including characteristic ring D loss and key diagnostic fragment ions, were performed according to the mass spectrometric fragmentation patterns and pathways proposed by Gorfer et al. (2023b). Since the modifications and substituents of the PBs identified in apple leaves showed consistent structural similarities with those previously characterized, they were systematically integrated into the MS database used for targeted screening. The PBs detected in this study comprised 14 NCCs, 6 DNCCs, 13 YCCs, 1 PiCC, Pheo *a*, and 1 hypermodified FCC (**Table 2**), all occurring as mixtures of stereoisomers, as expected. The most abundant PB detected in this study was NCC-644, previously reported from *Cercidiphyllum japonicum* (*Cj*-NCC-1) in pathogenically degreened apple leaves (Mittelberger et al., 2017) and in ripe apples and pears (Müller et al., 2007; Gorfer et al., 2022b). NCC-644 is a type-I phyllobilin characterized by a hydroxyethyl substituent at C-3 (R1), a methyl ester at C-8^3^ (R2), a carbonic acid at C-12^3^ (R3), and a vinyl group extending from ring D (C-18, R4, see **Table 2**). Several of the catabolites, including Pheo *a*, had previously been identified in apple peels (Pheo *a*, DNCC-632, YCC-642, NCC-644, DNCC-666, NCC-678, NCC-806, DNCC-828, NCC-840, DNCC-990, and NCC-1002) and leaves (NCC-628, PiCC-640, YCC-642, NCC-644, NCC-806, NCC-840, and NCC-1002) (Mittelberger et al., 2017; Gorfer et al., 2022b, 2022a, 2023b). The remaining PBs (23 in total) were previously detected in other plant species (DNCC-616, YCC-626, NCC-630, NCC-648, *hm*FCC-658, NCC-662, NCC-664, YCC-676, NCC-792, YCC-804, NCC-826, and YCC-838) or were tentatively identified using mass spectra patterns (YCC-612, YCC-628, DNCC-636, YCC-646, YCC-660, YCC-672, NCC-674, NCC-792, YCC-728, YCC-790, and YCC-1000).

**Figure 4:**
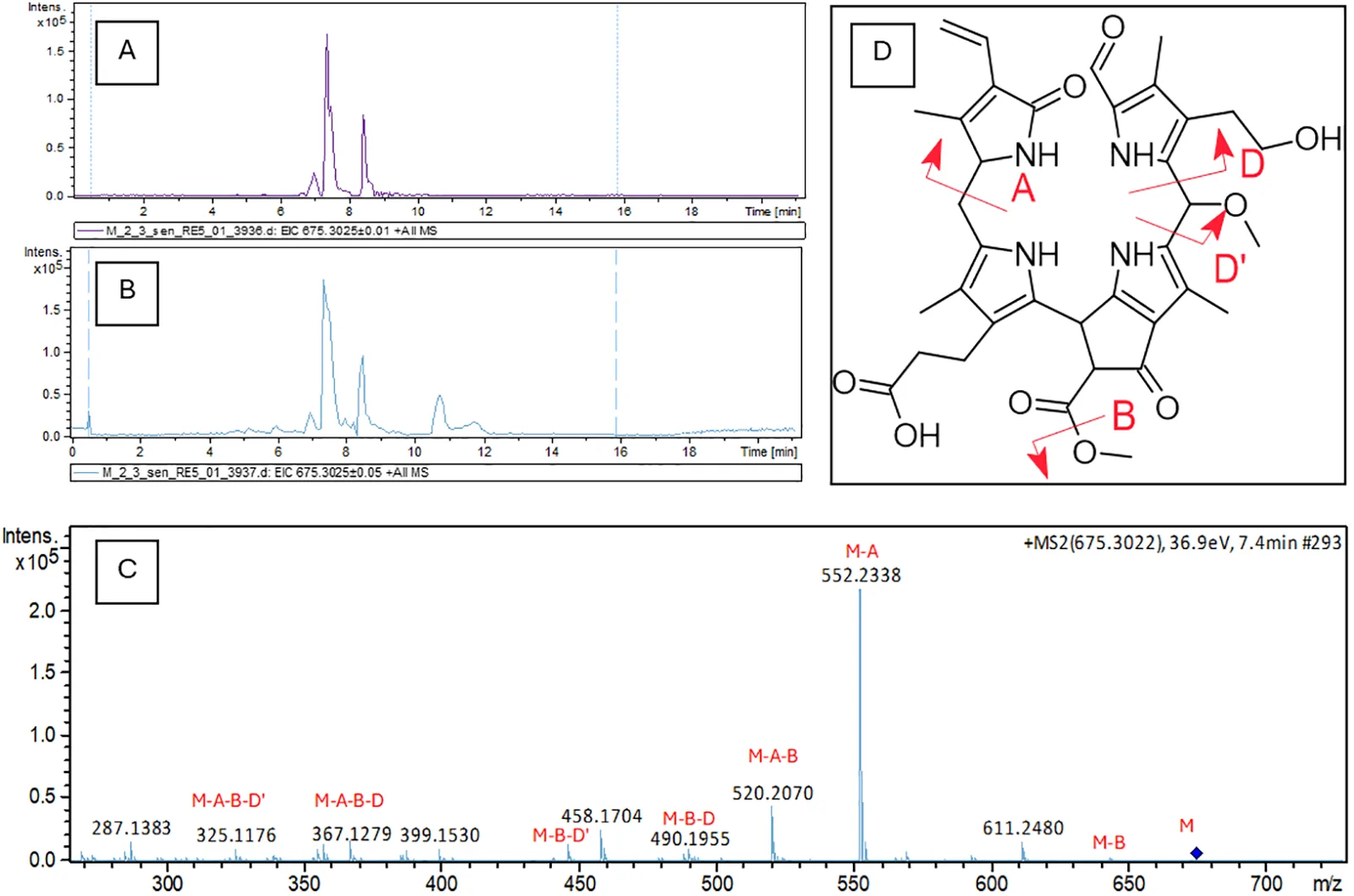
Extracted ion chromatogram obtained by full scan **(A)** and MS^2^ **(B)** of the putative novel 5-methoxy-phyllobilin detected in senescent apple leaves. The fragmentation pattern in MS^2^, along with the tentative structure and the fragmentation sites of the PB, is illustrated in **(C, D)**, respectively.

An intriguing new PB is NCC-674, with a base peak (*m/z*, [M+H]^+^) of 675.3025, consistent with the formula C_36_H_42_N_4_O_9_. Based on LC-MS data, including the fragmentation pattern, a tentative structure is proposed for this compound, with a methoxy group at C-5 on the PB backbone (**Figure 4**). However, alternative methoxy positions on the ring A–C core fragment cannot be unequivocally ruled out due to the low absolute intensity of the precursor ion (364 counts, **Supplementary Table 2**). For this reason, the compound has been assigned to Tier 3 (tentatively characterized). Furthermore, from a biosynthetic perspective, specific O-methyltransferases acting directly on PB backbones have not yet been characterized in *Malus* or the wider *Rosaceae* family (Hörtensteiner et al., 2019), leaving the exact enzymatic origin of this modification to be fully elucidated. Nevertheless, known fragmentation patterns of PBs support this proposed structure; in particular, the characteristic loss of ring D supports the conclusion that the methoxy group is located on the remaining fragment, most likely at C-5, as shown in **Figure 4**. Other typical fragments are illustrated in the figure. Notably, the LC-MS analysis also revealed features corresponding to the oxidized form YCC-672 (C_36_H_40_N_4_O_9_; **Table 2**, entry 20). In summary, the LC-MS method detected 36 of the 79 possible PBs across all samples and was used to create comprehensive PB profiles for comparing diseased and healthy (both green and senescent) leaves.

**Figure 5:**
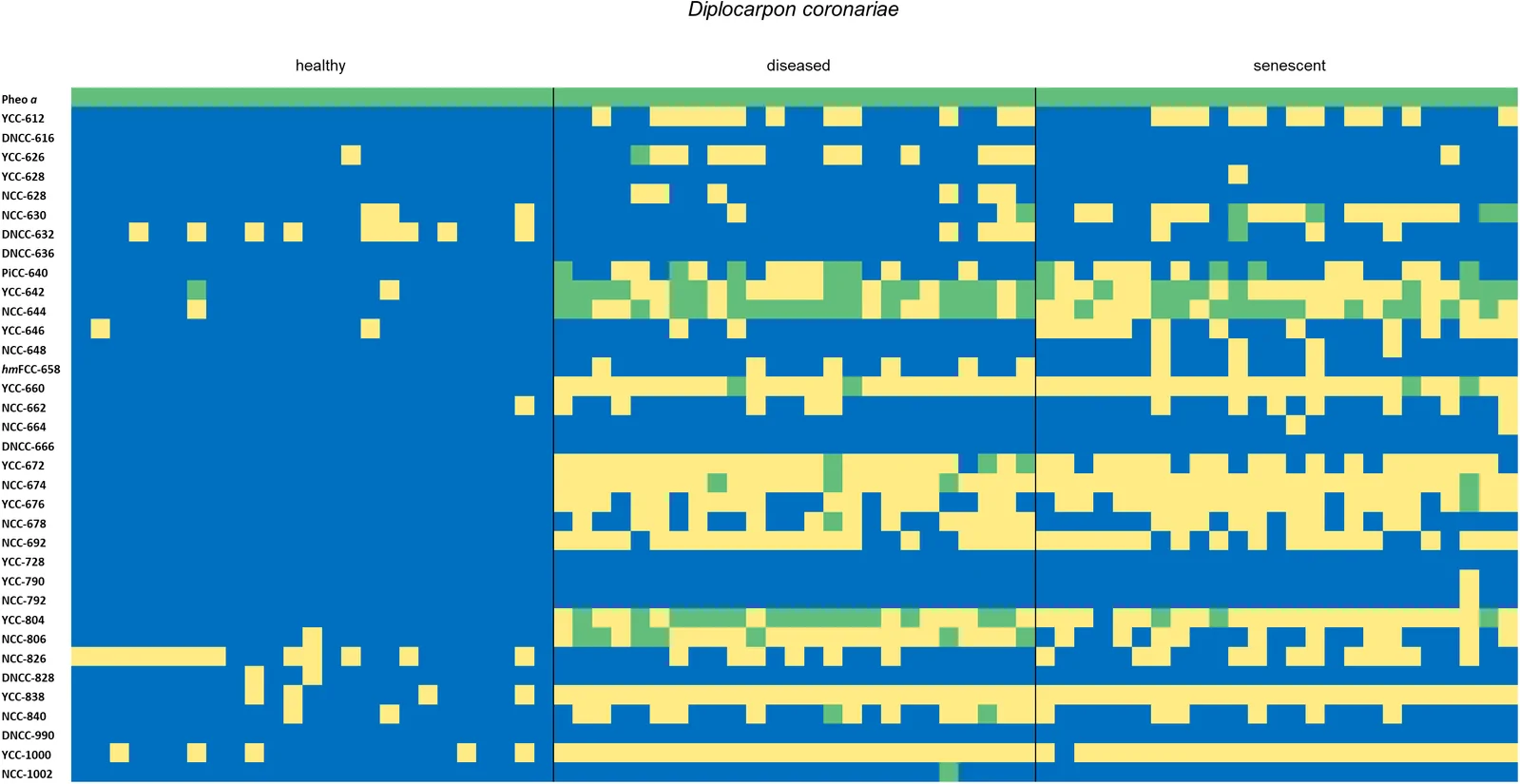
PB profiles in healthy, diseased (*Diplocarpon coronariae*), and senescent leaf samples from cv. Red Delicious. Each row represents an individual PB, and each column corresponds to a leaf sample (five leaves from five different plants per condition) PBs that were not detected are shown in blue; PBs detected only in FS are shown in yellow; and PBs detected in both FS and MS^2^ are shown in green. The categorical classification is based on a standardized extraction protocol using a fixed leaf surface area (two leaf discs per sample side) and a constant injection volume.

**Figure 6:**
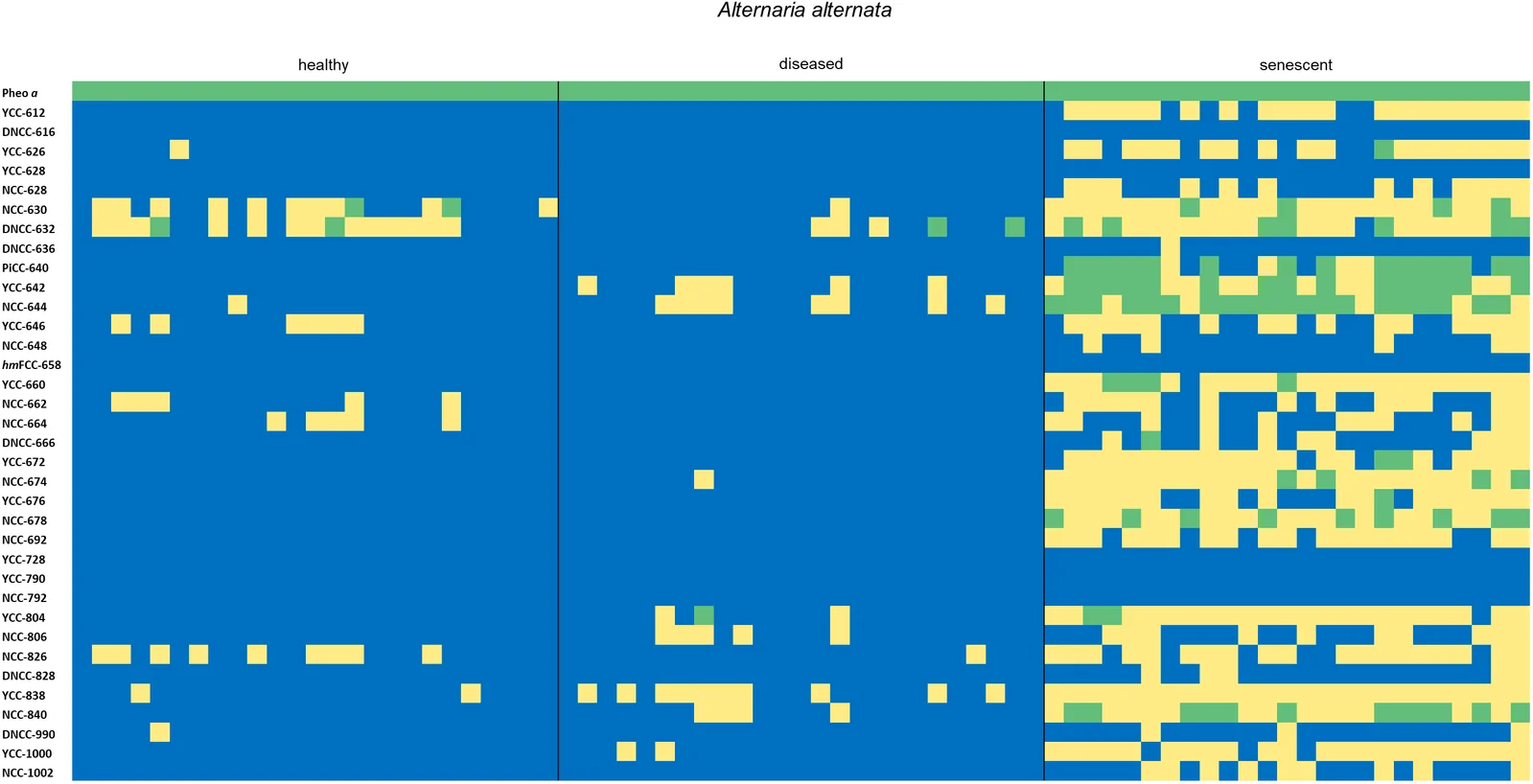
PB profiles in healthy, diseased (*Alternaria alternata*), and senescent leaf samples from cv. Golden Delicious. Each row represents an individual PB, and each column corresponds to a leaf sample (five leaves from five different plants per condition). PBs that were not detected are shown in blue; PBs detected only in FS are shown in yellow; and PBs detected in both FS and MS^2^ are shown in green. The categorical classification is based on a standardized extraction protocol using a fixed leaf surface area (two leaf discs per sample side) and a constant injection volume.

**Figure 7:**
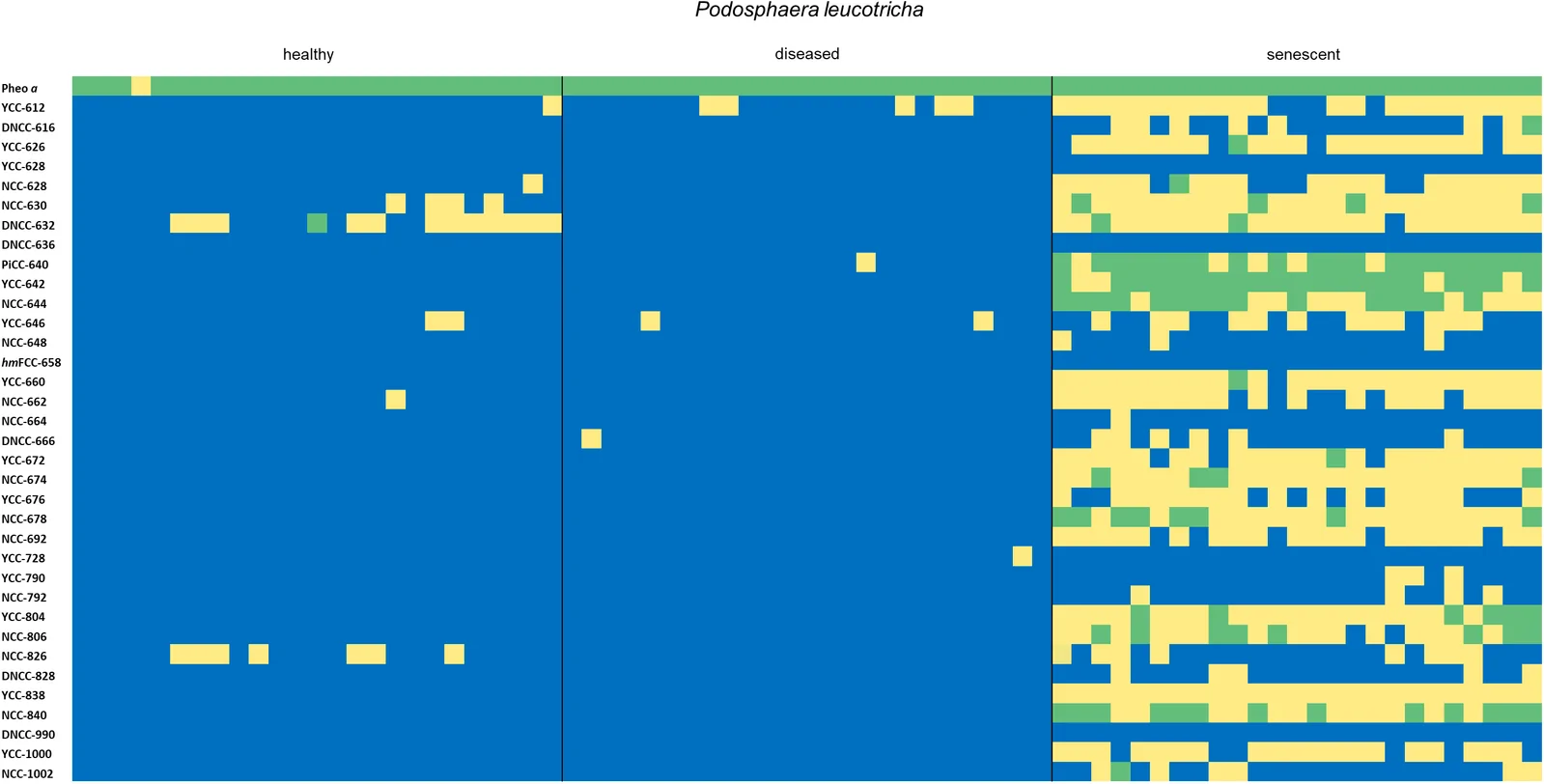
PB profiles in healthy, diseased (*Podosphaera leucotricha*), and senescent leaf samples from cv. Gala. Each row represents an individual PB, and each column corresponds to a leaf sample (five leaves from five different plants per condition) PBs that were not detected are shown in blue; PBs detected only in FS are shown in yellow; and PBs detected in both FS and MS^2^ are shown in green. The categorical classification is based on a standardized extraction protocol using a fixed leaf surface area (two leaf discs per sample side) and a constant injection volume.

**Figure 8:**
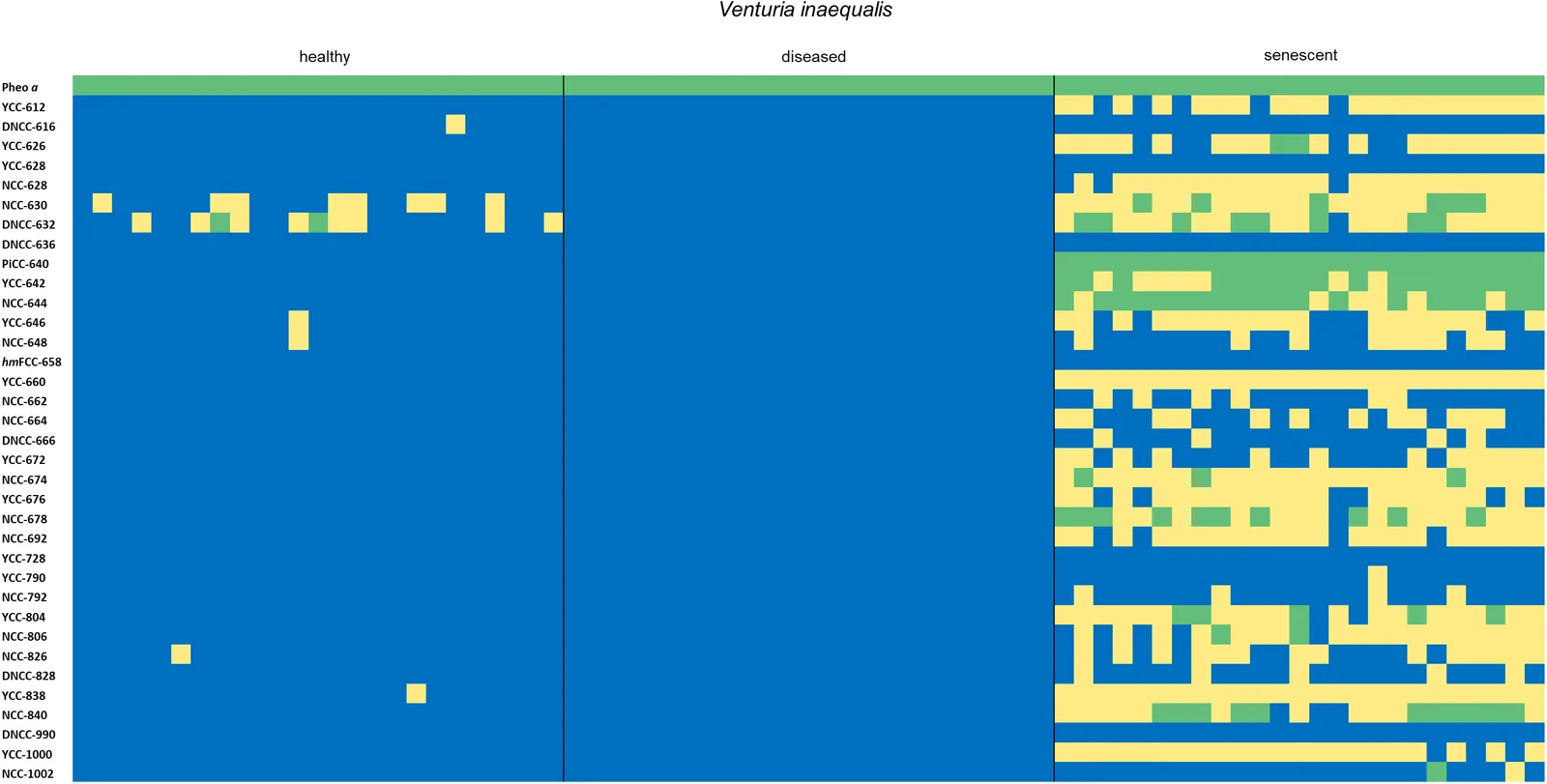
PB profiles in healthy, diseased (*Venturia inaequalis*), and senescent leaf samples from cv. Golden Delicious. Each row represents an individual PB, and each column corresponds to a leaf sample (five leaves from five different plants per condition) PBs that were not detected are shown in blue; PBs detected only in FS are shown in yellow; and PBs detected in both FS and MS^2^ are shown in green. The categorical classification is based on a standardized extraction protocol using a fixed leaf surface area (two leaf discs per sample side) and a constant injection volume.

PB profiles are illustrated in heatmaps showing the presence and distribution of the 36 PBs across healthy, diseased, and senescent apple leaves affected by all four pathologies (**Figures 5**–**8**). The visualization distinguished PBs according to their relative abundance in a semiquantitative way, with those detected in both full-scan and MS² modes classified as more abundant (or ‘major’), whereas those identified only in full-scan mode were considered less abundant (or ‘minor’). It must be emphasized that this classification is semiquantitative and serves primarily as an indicator of structural identification confidence rather than absolute chemical abundance. Because the analysis relied on data-dependent acquisition (DDA), the trigger of MS² spectra is subject to precursor competition. Consequently, highly intense ions may be operationally classified as ‘minor’ if they fail to undergo fragmentation.

In leaves of cv. Red Delicious infected with *D. coronariae*, where Chl degradation was pronounced and extensive chlorosis was evident, the highest number of PBs was identified, with several PBs detected in both full-scan and MS² modes (**Figure 5**). To assess the alignment between the profiles, the Jaccard similarity index was calculated as the percentage of shared PBs relative to the total number of distinct catabolites detected within each sample group (representing the ratio of shared PBs to the total group pool). A total of 28 PBs were detected in senescent leaves, 23 of which were also present in the infected samples. In cv. Red Delicious, the baseline overlaps between healthy green leaves, and the senescent reference was 41% (12 out of 29 PBs). Crucially, upon infection with *D. coronariae*, this overlap significantly increased to 77% (23 out of 30 PBs shared with the senescent reference). The concentration of several PBs was even higher in infected leaves, as evidenced by their detection in both full scan and MS² modes, compared with senescent leaves (e.g., YCC-642, NCC-644, YCC-804, or NCC-806); this observation could be related to the incomplete Chl breakdown in senescent leaves from this study group, as detailed above. Senescent leaves contained several ‘major’ PBs, including NCC-630, DNCC-632, PiCC-640, YCC-642, NCC-644, YCC-660, NCC-674, YCC-676, and YCC-804. The remaining Chl catabolites were ‘minor’ PBs, including YCC-612, YCC-626, YCC-628, YCC-646, NCC-648, *hm*FCC-658, NCC-662, NCC-664, YCC-672, NCC-678, NCC-692, YCC-790, NCC-792, NCC-806, NCC-826, YCC-838, NCC-840, and YCC-1000. In diseased leaves, ‘major’ PBs were: YCC-626, NCC-630, PiCC-640, YCC-642, NCC-644, YCC-660, YCC-672, NCC-674, NCC-678, YCC-804, NCC-806, NCC-840, and NCC-1002. ‘Minor’ PBs were: YCC-612, NCC-628, DNCC-632, YCC-646, *hm*FCC-658, NCC-662, YCC-676, NCC-692, NCC-826, YCC-838, and YCC-1000. Healthy, green leaves are not expected to contain PBs; nevertheless, a few ‘minor’ PBs (YCC-626, NCC-630, DNCC-632, NCC-644, YCC-646, NCC-662, NCC-806, NCC-826, DNCC-828, YCC-838, NCC-840, and YCC-1000) and one ‘major’ PB (YCC-642), in addition to Pheo *a*, were detected, consistent with previous studies. Notably, the number of detected PBs correlated with chlorosis. Green, healthy leaves showed minimal PB accumulation and no signs of chlorosis, whereas infected leaves exhibited both elevated PB levels and premature yellowing. This suggests that *D. coronariae* infection engages the PaO/PB pathway, as observed for phytoplasma infections in apple and apricot (Mittelberger et al., 2017). The similarity between PB profiles in infected and naturally senescent leaves further supports this interpretation, confirming a general involvement of the PaO/PB pathway in the development of chlorotic symptoms during infection. Furthermore, the late-season occurrence of *D. coronariae* infection may have coincided with the plant’s transition toward winter dormancy.

In the *A. alternata* infections, chlorotic symptoms were less evident (**Table 1**). Indeed, *A. alternata*-infected leaves contained fewer PBs than leaves infected with *D. coronariae*, despite a higher number of PBs being detected in senescent leaves. Specifically, 30 PBs were identified in senescent leaves, compared with only 12 and 11 in symptomatic and healthy leaves, respectively (**Figure 6**). Healthy green leaves shared 37% of PBs (11 out of 30 PBs with the senescent reference), while infected leaves shared 40% (12 out of 30). ‘Major’ PBs included YCC-626, NCC-630, DNCC-632, PiCC-640, YCC-642, NCC-644, YCC-660, DNCC-666, YCC-672, NCC-674, YCC-676, NCC-678, YCC-804, and NCC-840. ‘Minor’ PBs were YCC-612, NCC-628, DNCC-636, YCC-646, NCC-648, NCC-662, NCC-664, NCC-692, NCC-806, NCC-826, DNCC-828, YCC-838, DNCC-990, YCC-1000, and NCC-1002, indicating a shift in the relative abundance pattern of PBs compared with *D. coronariae*. In *A. alternata*-infected leaves, DNCC-632 and YCC-804 were ‘major’ PBs, whereas NCC-630, YCC-642, NCC-644, NCC-674, NCC-806, NCC-826, YCC-838, NCC-840, and YCC-1000 were ‘minor’. Even though this method does not allow reliable quantification, the semiquantitative analysis suggests frequent shifts in the abundance pattern of PBs, as also observed in healthy leaves. NCC-630 and DNCC-632 were ‘major’ PBs in cv. Golden Delicious, while YCC-626, NCC-644, YCC-646, NCC-662, NCC-664, NCC-826, YCC-838, and DNCC-990 were ‘minor’. Unlike *D. coronariae*, *A. alternata* caused necrotic lesions without inducing visible chlorosis. Accordingly, the PB profile observed in affected tissue closely resembled that of healthy leaves and differed markedly from the profile characteristic of senescent leaves (**Figure 6**). The lesions are caused by mycotoxins produced by *A. alternata*, which play important roles in fungal colonization and disease induction. Mycotoxin action sites include the plasma membrane and chloroplasts (Wolpert et al., 2002). It has been shown that necrotic lesions developing following treatment with mycotoxins are similar to those produced by the pathogen (Cao et al., 2024). Therefore, although the fungus affects the plant through its mycotoxins, infection of the host plant did not significantly affect the PB profile in apple leaves. Thus, although the pathogen is known to induce cell membrane damage, disrupt cell wall proteins, trigger reactive oxygen species production, and increase H_2_O_2_ accumulation, leading to cell death (Meena et al., 2016), the present study found no evidence for the engagement of the PaO/PB pathway or the accumulation of its downstream products. However, it must be noted that our targeted screening utilized an inclusion list of 79 known chlorophyll catabolites. Consequently, this experimental design cannot rule out the potential presence of novel, atypical, or pathogen-modified PBs arising from non-canonical degradation routes.

Cv. Gala leaves infected with *P. leucotricha* showed a similar PB profile to *A. alternata*-infected cv. Golden Delicious leaves but contained fewer PBs (six), including YCC-612, PiCC-640, YCC-646, DNCC-666, and YCC-728. Senescent leaves of cv. Gala displayed a comparable PB composition to the other groups, with a total of 31 catabolites (**Figure 7**). ‘Major’ PBs included DNCC-616, YCC-626, NCC-628, NCC-630, DNCC-632, PiCC-640, YCC-642, NCC-644, YCC-660, YCC-672, NCC-674, NCC-678, YCC-804, NCC-806, NCC-840, and YCC-1002. ‘Minor’ PBs included YCC-612, YCC-646, NCC-648, NCC-662, NCC-664, DNCC-666, YCC-676, NCC-692, YCC-728, YCC-790, NCC-792, NCC-826, DNCC-828, YCC-838, and YCC-1000.

Eight PBs were detected in healthy leaves, clearly showing that infection did not have a substantial influence on the PB profile. Notably, DNCC-632 and NCC-630 were detected in healthy leaves but were not detected in infected leaves. Similar to *A. alternata*, symptoms did not include extensive chlorosis but were characterized by leaf wilting and a grayish-white powdery mycelium on the abaxial surface. The fungus *P. leucotricha* is an obligate parasite that enters plant cells through haustoria. When apple leaves are infected, the pathogen attacks the cell wall and simultaneously triggers relevant transduction pathways, such as reactive oxygen species burst and protein kinase activity, leading to the biosynthesis and signaling of plant hormones. These pathways further activate secondary and tertiary regulatory networks, including transcription factors, which activate or inhibit the expression of downstream genes essential for plant function (Bowen et al., 2011). As evident from this study, there is no metabolic evidence of PaO/PB pathway engagement in the hosts’ response to infection with *P. leucotricha*. Nevertheless, this observation is limited by the 79 canonical PBs monitored using our targeted approach, leaving open the possibility that non-canonical or structurally modified derivatives may be present but remain undetected with our method.

Finally, in *V. inaequalis* infected cv. Golden Delicious leaves, no PBs, apart from Pheo *a*, were detected. In contrast, a typical PB profile was found in senescent leaves, with a total of 30 catabolites. While healthy leaves shared 23% of PBs (7 out of 31), this overlap dropped to just 3% in diseased leaves (1 out of 30). This slight decrease clearly shows that infection did not have a substantial inductive influence on the PB profile. Again, eight PBs were found in healthy samples (**Figure 8**). During infection, *V. inaequalis* caused no obvious damage to the host throughout stroma development, apart from breaching the cuticle. Only after sporulation do the epidermal cells underlying the stroma undergo a progressive depletion of plastids and cytoplasm, accompanied by increasing vacuolation, ultimately leading to necrosis, which is possibly affected by partial cell wall degradation late in the infection cycle. Enzymes are expressed only late in infection, during host cell wall degradation; therefore, enzymes are unlikely to play a major role in nutrient acquisition during the establishment of infection (Bowen et al., 2011). Symptoms included olive-green to brown lesions on leaves, often surrounded by distinct chlorotic halos. Even though the lesions were excised during sampling, no PBs were detected. There is evidence that, similar to other biotrophic phytopathogens, *V. inaequalis* influences hormone levels at infection sites; specifically, cytokinin accumulation results in the translocation of nutrients to the fungus (Walters and McRoberts, 2006; Bowen et al., 2011). *V. inaequalis* infection is the most prevalent and relevant disease in commercial apple production. This study provides no indication of engagement of the PaO/PB pathway in the analyzed lamina tissue at the specific developmental stage sampled, but cases with more extensive chlorotic halos around the lesions warrant further investigation. Additionally, future studies should consider an untargeted approach, as our findings are based on the 79 PBs present in the inclusion list.

To the best of our knowledge, this study provides the most comprehensive description to date of phyllobilins (PBs) in senescent and infected apple tissue. Using the recently published high-resolution LC–MS method developed by Gorfer et al. (2022a), with an updated inclusion list comprising 79 known and putative PBs, we detected a remarkable total of 36 PBs (including Pheo *a*) across all samples. In previous studies, typically only a single-digit number of PBs has been reported per plant species, underscoring the utility of this mass spectrometry method for future PB profiling.

By enabling the accurate detection of PBs at low concentrations, this approach allowed us to follow Chl breakdown in both green and pathogen-infected leaves. PBs were present in green and infected leaves, although generally in limited diversity and at low concentrations. A notable exception was *D. coronariae*-infected leaves of cultivar ‘Red Delicious’, which showed a PB profile comparable to that of senescent leaves. *D. coronariae* caused extensive leaf yellowing, whereas visual signs of chlorosis were limited or absent in the other three pathogens, consistent with PB profiles more similar to those of green leaves. Not unexpectedly, these findings point to a correlation between PB accumulation and leaf chlorosis, supporting the conclusion that the PaO/PB pathway is generally engaged during infections that induce chlorosis, such as those caused by *D. coronariae*, *Candidatus* Phytoplasma mali, and *Candidatus* Phytoplasma prunorum (Mittelberger et al., 2017). In contrast, during infections, in which leaves remain green, the PaO/PB pathway does not appear to be activated.

However, a methodological limitation should be considered when comparing pathogen-specific effects. Because of practical agronomic availability, different cultivars were used (cv. Red Delicious for *D. coronariae*, cv. Golden Delicious for *A. alternata* and *V. inaequalis*, and cv. Gala for *P. leucotricha*). Healthy leaves already showed cultivar-dependent PB differences, with NCC-630 and DNCC-632 prominent only in cv. Golden Delicious, indicating an influence of genetic background on Chl catabolism. Sampling time likely further contributed, as the majority of infected samples were collected in July, whereas *D. coronariae* samples were obtained in September, coinciding with seasonal PB accumulation. Although healthy and diseased leaves were collected on the same day, the strong increase in PBs observed in *D. coronariae*-infected tissues likely reflects both pathogen effects and presenescence processes.

Another limitation is the use of natural infections achieved by withholding fungicides, which limits control over infection timing, stage, and severity. This is evident for *V. inaequalis*, where no downstream PBs were detected. This may reflect the intermediate lesion stage sampled rather than an intrinsic inability to activate the PaO/PB pathway.

Further investigations are needed to address these aspects and elucidate the role of the PaO/PB pathway in chlorotic stress responses within plant defense mechanisms.

## Data Availability

The raw data supporting the conclusions of this article will be made available by the authors, without undue reservation.
